# EFForTS-LGraf: A landscape generator for creating smallholder-driven land-use mosaics

**DOI:** 10.1371/journal.pone.0222949

**Published:** 2019-09-27

**Authors:** Jan Salecker, Claudia Dislich, Kerstin Wiegand, Katrin M. Meyer, Guy Pe´er

**Affiliations:** 1 Ecosystem Modelling, Faculty of Forest Sciences and Forest Ecology, University of Goettingen, Goettingen, Germany; 2 Centre of Biodiversity and Sustainable Land Use (CBL), University of Goettingen, Goettingen, Germany; 3 Synthesis Centre (sDiv) of the German Centre for Integrative Biodiversity Research (iDiv) Halle-Jena-Leipzig, Leipzig, Germany; 4 UFZ - Helmholtz Centre for Environmental Research, Dept. Economics and Dept. Ecosystem Services, Leipzig, Germany; 5 University of Leipzig, Leipzig, Germany; Indiana State University, UNITED STATES

## Abstract

Spatially-explicit simulation models are commonly used to study complex ecological and socio-economic research questions. Often these models depend on detailed input data, such as initial land-cover maps to set up model simulations. Here we present the landscape generator EFFortS-LGraf that provides artificially-generated land-use maps of agricultural landscapes shaped by small-scale farms. EFForTS-LGraf is a process-based landscape generator that explicitly incorporates the human dimension of land-use change. The model generates roads and villages that consist of smallholder farming households. These smallholders use different establishment strategies to create fields in their close vicinity. Crop types are distributed to these fields based on crop fractions and specialization levels. EFForTS-LGraf model parameters such as household area or field size frequency distributions can be derived from household surveys or geospatial data. This can be an advantage over the abstract parameters of neutral landscape generators. We tested the model using oil palm and rubber farming in Indonesia as a case study and validated the artificially-generated maps against classified satellite images. Our results show that EFForTS-LGraf is able to generate realistic land-cover maps with properties that lie within the boundaries of landscapes from classified satellite images. An applied simulation experiment on landscape-level effects of increasing household area and crop specialization revealed that larger households with higher specialization levels led to spatially more homogeneous and less scattered crop type distributions and reduced edge area proportion. Thus, EFForTS-LGraf can be applied both to generate maps as inputs for simulation modelling and as a stand-alone tool for specific landscape-scale analyses in the context of ecological-economic studies of smallholder farming systems.

## Introduction

Land-use change was highlighted as one of the most important anthropogenic impacts on ecosystems [1]. Agent-based models (ABMs) are widely applied to study the human perspective of land-use change (MAS/LUCC models) and social-ecological systems (SESs), addressing the tight inter-coupling between ecological and socio-economic processes [2–4]. Recent spatially-explicit simulation models in land-use science typically incorporate environmental heterogeneity by modelling the spatio-temporal distribution and dynamics of land-cover types (e.g. [5, 6]).

However, environmental data, such as land-cover maps, can often not be obtained at the necessary level of detail. This problem is especially prevalent in tropical regions, where constant cloud coverage limits remote sensing data usability. In such cases, as well as for systematic research analyses, it may be necessary to artificially generate land-cover maps that approximate reality. For this purpose, landscape generators (also called landscape simulators) have been developed [7]. A typical landscape generator creates landscapes consisting of a grid of cells, where each cell is assigned a given land-cover type. Landscape generators can also be used to systematically generate many similar land-cover maps and thereby allow for scenario-based control of landscape characteristics. Moreover, the option of producing a range of (also non-realistic) landscapes, e.g. by setting population density or field sizes to unrealistically high values, is an important tool for testing potential scenarios and improving the understanding of model processes.

Two approaches are known for landscape generators: pattern-based landscape generators and process-based landscape generators [7]. Many pattern-based landscape generators are based on artificial (e.g. fractal) algorithms with relatively low model complexity and only a small number of parameters. The resulting landscapes are often known as neutral landscapes [8, 9]. However, real world land-cover patterns are often the result of historical land-use change by human interaction with the landscape and as such a result of the processes that shape these landscape. Furthermore, the outcomes of these interactions often result in spatial patterns that differ substantial from those shaped by neutral processes. Thus, in contrast to pattern-based landscape generators, process-based generators try to simulate the processes that lead to the pattern [10]. The process-based approach can be more costly regarding the number of parameters and model complexity. However, parameters often have an empirical meaning and can be measured with surveys allowing for creation of artificial landscapes that mimic properties of real world landscapes of a certain study area.

Two key processes driving anthropogenic land-use changes are the rapid expansion of roads [11, 12] and the related expansion and intensification of agricultural fields [13]. Models that incorporate these processes into models of landscape design, such as DYPAL [14] and G-RaFFe [15], have been shown to successfully produce realistic ranges of landscape characteristics for anthropogenically-altered landscapes. However, for ABMs that explicitly incorporate the human dimension, a realistic land-cover map alone might not be sufficient. For example, EFForTS-ABM, an agent-based model studying land-use change in agricultural landscapes dominated by smallholders in Indonesia, requires additional information regarding fields (as agricultural units) and land ownership, because smallholder households are modelled as agents that own these fields [16]. The determination of fields and allocation to specific households cannot be done using remote sensing data and is difficult to obtain using existing landscape generator approaches. In consequence, the human perspective is often lacking completely in landscapes used for ecological research (including modelling), and in fact, forming one of the key gaps between ecological and socio-economic research.

Thus, an extension of the process-based approach to landscape generation is needed that overcomes this mismatch and incorporates the human dimension of landscape ownership and management. Such an extension would not only provide spatially explicit land-use maps, but would also deliver information on properties of smallholder farmers and land ownership and thus enable users to inform agent-based models, to study decision-making, ecosystem functioning trade-offs, and evaluate agricultural policies in more detail.

Here, we present a new model, EFForTS-LGraf, for creating land-cover maps that represent agricultural areas dominated by smallholders. We focus on smallholders because they comprise a large proportion of farmers in most parts of the world. The model allows for flexible parameterization of the main processes shaping these landscapes, i.e. road creation and field establishment by smallholder farming households. The model algorithms follow the assumption that the creation and expansion of agricultural land is connected to road establishment, as has been reported in several case studies (e.g., [17–19]). We build upon G-Raffe, an existing landscape generator that simulates the process of field establishment along roads [15] but does not explicitly incorporate the household dimension. We extended the algorithms of G-Raffe by using an agent-based modelling approach and introducing smallholder farming households that have a specific home-base and a given household size.

We present a detailed model description of EFForTS-LGraf, using the ODD (Overview, Design concepts and Details) protocol for describing agent-based models [20, 21] and the ODD+D (ODD + Decision) extension of the protocol for describing agent-based models that involve human decisions [22]. We take smallholder farming in Jambi province, Indonesia, as an example to demonstrate model applications using three approaches (Details, see Section Scenarios and parameterization): (1) We use a sensitivity analysis to quantify effects of EFForTS-LGraf model parameters on properties of the landscapes generated. (2) We compare a classified land-use map from our study region to the landscapes generated with EFForTS-LGraf. (3) In an applied case study we investigate effects of household consolidation and specialization on landscape patterns. All approaches are based on quantification of landscape characteristics via landscape metrics.

## Model description

EFForTS-LGraf was implemented in NetLogo 6.0.2. The EFForTS-LGraf model and a manual on parameterization and execution of EFForTS-LGraf is provided in a publicly available online repository (https://doi.org/10.5281/zenodo.2677496). This repository also includes R scripts to reproduce our model analyses, corresponding output files and plots.

### Overview

#### Purpose

The general goal of the EFForTS-LGraf landscape generator is to create artificial maps of landscapes that are dominated, or strongly shaped, by agricultural activities. The grid-based maps include fields of various sizes and different crop types and other potential land-cover types as desired. These other potential land-cover types, such as forest, grassland, water bodies or degraded land are grouped into a single land-cover type (here, we used the general term ‘others’). In addition to fields, the model considers land ownership by assigning each field to a farming household agent. Artificial land-cover maps produced by EFForTS-LGraf can be used as a template, or input, for other models which, for example, can simulate the effects of land-use types on ecological and/or economic functions. The resulting maps may also be used as a starting point to analyse how farmer decisions alter land-use and shape land-use changes.

#### Entities, state variables and scales

The simulated landscape comprises several spatial units: cells, fields, households and the landscape. Roads are a non-spatial unit of EFForTS-LGraf as they are similar to polylines in a GIS context. Cells are the smallest spatial unit of EFForTS-LGraf. The landscape consists of a regular grid of these cells. The cell size can be set by the user and should approximately correspond to the smallest size of fields in the landscape. The extent of the landscape can also be set by the user. Cell attributes include its land-cover type, e.g. ‘others’ or ‘field’, and the household the cell belongs to (if any). Moreover, each cell has the attribute of whether it contains an intersecting road or not. The fields are composed of one or several contiguous cells that have all the same crop type and belong to (or are managed by) the same household. Each household has a home-base cell and owns one or several fields that do not need to occur next to each other. The household is represented by an agent that is establishing fields close to the household’s home-base cell during the simulation process.

#### Process overview and scheduling

**Fig 1:**
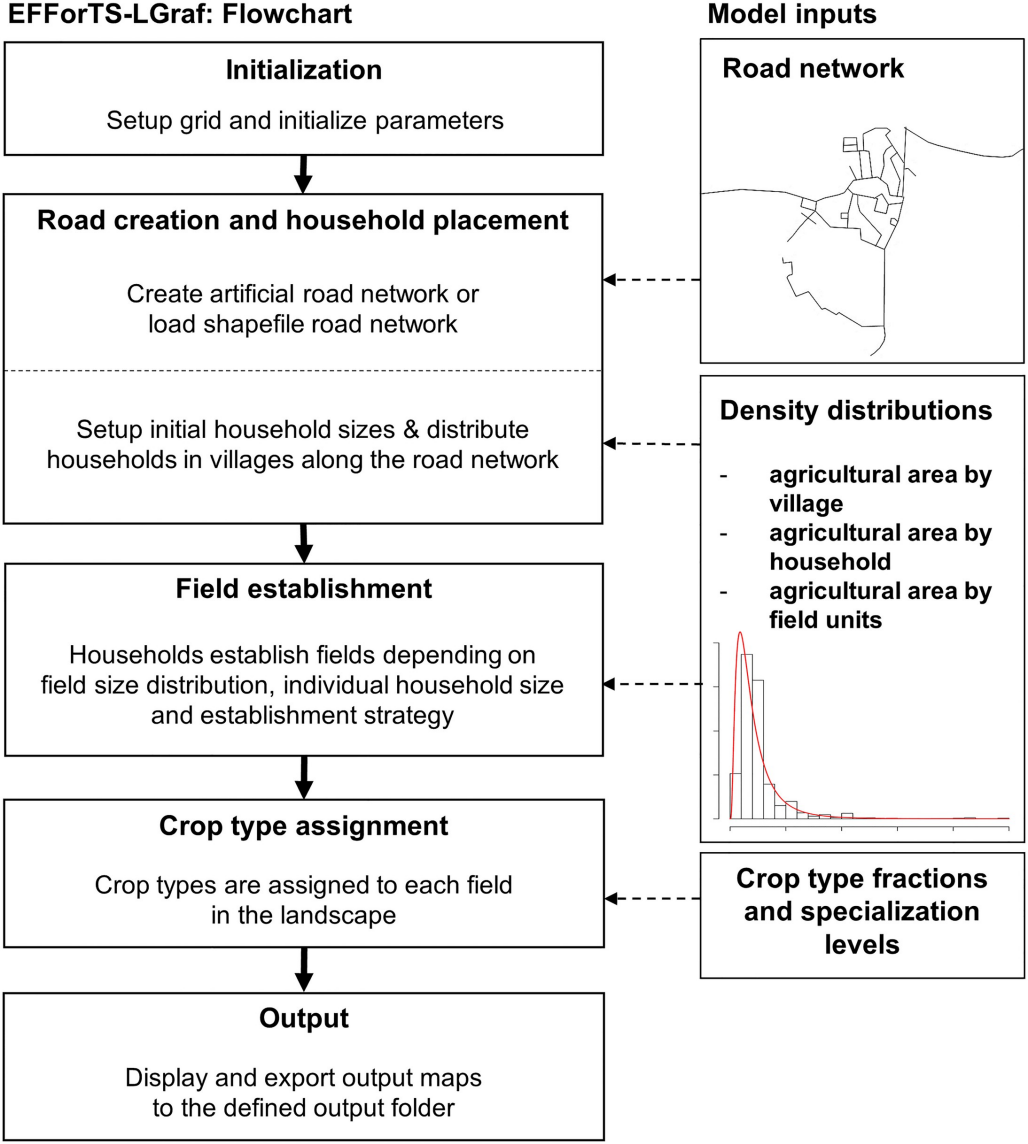
EFForTS-LGraf flowchart including process flow of main model processes and model inputs.

**Fig 2:**
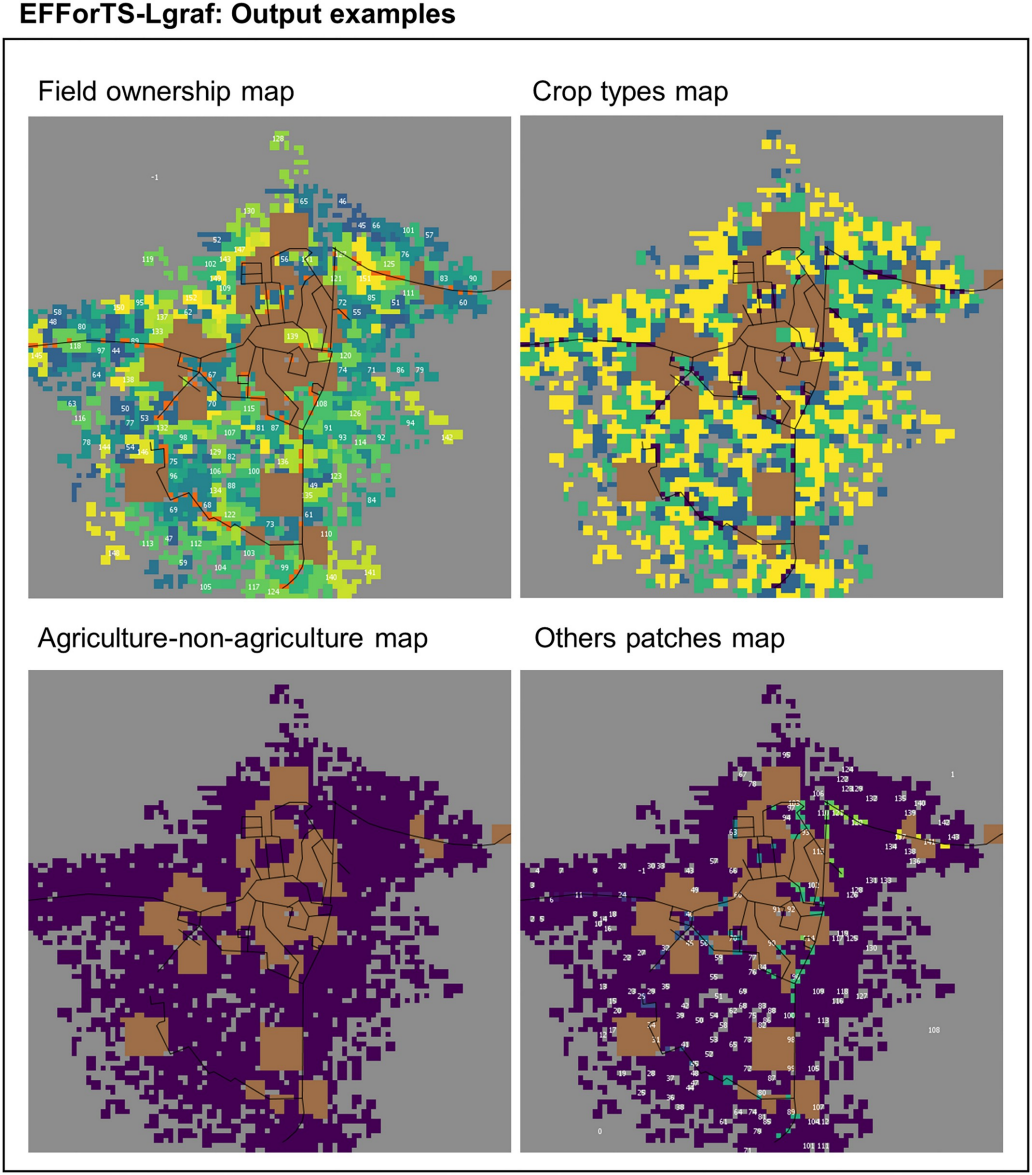
Output map examples of EFForTS-LGraf. All maps include patches of inaccessible area (brown color) and roads (black lines). In the field ownership map (upper left), hues indicate field owners. In the crop type map (upper right) colors indicate fields with different crops. The agriculture-non-agriculture map (lower left), is a binary map that differentiates agricultural cells (purple) from other cells (grey). The ‘others’ patches map (lower right) is similar to the agriculture-non-agriculture map but shows each separate patch of class ‘other’ in another color.

EFForTS-LGraf is initialized with a landscape completely covered by cells of the general class ‘others’ (Fig 1). Within the procedure “Road creation and household placement” a network of roads is established either artificially or taken as input from a realistic road map (see Road creation and household placement in Section Submodels). The number of households in the landscape is determined and households are placed onto the map by assigning home-base cells. All home-base cells belong to the road network, i.e. home-base cells always have a road intersecting them. The spatial distribution of households may be completely random or aggregated in villages, depending on the village size distribution. In the “Field establishment” procedure, the households establish fields close to their home-base cell. This procedure is designed such that the resulting frequency distribution of field sizes as well as the resulting frequency distribution of household areas approximate the expected distributions set by the input parameters (see Field establishment in Section Submodels). Thereafter, the procedure “Crop type assignment” assigns crop types to the established fields based on input parameters such as fractions of crop types and specialization degree (see Crop type assignment in Section Submodels). Finally, different maps of the simulated landscape are produced as model output, such as land-cover map and land-ownership map (see Section Output data and Fig 2).

### Design concepts

#### Theoretical and empirical background

The main processes in EFForTS-LGraf build upon the assumption that households settle along roads and establish fields in close vicinity to their home. Such patterns have been reported in several case studies (e.g., [17–19]) and have successfully been implemented in other landscape generators [15]. EFForTS-LGraf does not incorporate any temporal dimension. Instead of simulating the process of land-use change over time explicitly, distributions of household and field sizes are used to create a landscape at one specific point in time. The model does not incorporate any environmental heterogeneity and all considered crop types are equally suitable throughout the landscape. However, the amount and distribution of crop types can be controlled by adjusting landscape proportions and household specialization levels for each crop type.

#### Individual decision-making

Households establish fields based on several decisions. They search for an unoccupied cell in the vicinity of their home-base and try to establish a field of a given size there. If this action is not successful, e.g. because the area between cells that are already occupied is too small, they continue to try in a different location. Finding an unoccupied cell depends on the current field establishment strategy (for Details see Field establishment in Section Submodels). If a household fails to establish a field for a given number of times under the current strategy, it switches the establishment strategy. Current establishment strategies include searching for unoccupied cells in vicinity of the home-base, in vicinity of already established fields, in neighboring cells of class ‘others’ with gradually increasing search radius, and searching for unoccupied cells that are surrounded only by unoccupied cells. If the attempt to establish a field of the given size fails for all potential strategies, a new field size is drawn from the field size distribution.

#### Individual sensing

As in reality, households are assumed to have full knowledge on land-use types and ownership of all cells in the landscape. A household cannot expand a new field into an already existing field, whether it is owned by a different household or by the same household.

#### Interaction

Households interact indirectly by land ownership, e.g. one household cannot expand a new field into the field of a different household.

#### Heterogeneity

Household agents are heterogeneous in their expected and realized household area. Households also differ in number, size and shape of established fields. Additionally, households may cultivate different proportions of crop types and some households may also specialize on one specific crop type. Households are aggregated in villages and have different initial home-base positions. The initial size of the village and home-base position may affect field establishment by the household, which may for instance result in higher distances between home-base and fields (in densely populated areas). These aspects of heterogeneity are mostly imposed by external inputs that determine the overall distribution of field sizes, household areas and village areas.

#### Stochasticity

The location of household home-base cells along the road network is random. However, aggregation of households at certain locations can be controlled by providing a village size distribution (see Road creation and household placement in Section Submodels). Field sizes and household areas are drawn from a given probability distribution and are therefore also stochastic. In addition, in the case that the user chooses to create the road network within EFForTS-LGraf, the algorithm for road generation randomly assigns the location, direction and length of each road segment (see Section Submodels).

#### Observation/Emergence

The spatial arrangement of fields is an emergent property of the model and will thus differ between simulations. The emerging patterns can be compared to real maps or used to generate a set of virtual land-use maps. Aggregated model outputs that can be generated from the maps include spatially implicit data such as maximum distance between roads and fields.

### Details

#### Input data and initialization

A potential external input for EFForTS-LGraf is a map of an existing road network (option real-road-map). Most of the model parameters (Table 1) can be estimated from empirical studies or remote sensing data. In addition to model parameters (numeric), switches (logical, categorical) allow turning on and off certain model processes (Table 1). Several distribution parameters affect model behaviour and outcomes: (1) A household size distribution, from which the total agricultural area of each household is sampled during model initialization. (2) A village size distribution, from which the village sizes (number of households per village) are sampled during model initialization. (3) A field size distribution, from which the areas of the single field units are sampled during field establishment. (4) Inaccessible area distribution, from which areas of each inaccessible area patch are sampled during model initialization. Different distribution types are possible and include constant, uniform, normal and log-normal shapes. Additionally, road parameters, crop type proportions, crop type specialization levels and field establishment strategies need to be set.

**Table 1:** EFForTS-LGraf model parameters.

id	Name on GUI	Unit	Description
*t*_s_	setup-type	[-]	*households*: number of villages and agricultural area are approximated by providing a fixed number of households; *villages*: number of households and agricultural area are approximated by providing a fixed number of villages; *area*: number of households and villages are approximated by providing the total agricultural area
*n*_s,h_	number-of-farmers	[-]	number of farming households in landscape
*n*_s,v_	number-of-villages	[-]	number of villages in landscape
*n*_s,a_	prop-agricultural-area	[-]	proportion of agricultural area in landscape
*n*_s,c_	households-per-cell	[-]	maximum number of household home-bases in one cell
*Seed*_s_	rnd-seed	[-]	random seed of the simulation, only used when *rep*_s_ is true
*rep*_s_	reproducable?	[-]	if true, the user-set random seed *Seed*_s_ is used
*w*_s_	width	cell	width of the landscape grid
*h*_s_	height	cell	height of the landscape grid
*c*_s_	cell-length-meter	cell	side length in meter of one cell of the landscape grid
*t*_r_	road-type	[-]	type of road algorithm (*shapefile, artificial.perlin* or *artificial.graffe*)
*i*_r,shp_	road-map-nr	[-]	number of road map file (only used when *t*_r_ is *shapefile*)
*n*_r,art_	total-road-length	cell	total number of road cells in landscape (only used when *t*_r_ is *artificial.perlin* or *artificial.graffe*)
*m*_r,art_	min-dist-roads	cell	minimum distance [cells] between two roads (only used when *t*_r_ is *artificial.perlin* or *artificial.graffe*)
*p*1_r,perl_	perlin-octaves	cell	octaves parameter for the perlin algorithm (only used when *t*_r_ is *artificial.perlin*)
*p*2_r,perl_	perlin-persistence	cell	persistence parameter for the perlin algorithm (only used when *t*_r_ is *artificial.perlin*)
*p*3_r,perl_	cone-angle	cell	cone-angle parameter for the perlin algorithm (only used when *t*_r_ is *artificial.perlin*)
*p*4_r,perl_	dist-weight	cell	distance versus elevation weighting for the perlin algorithm (only used when *t*_r_ is *artificial.perlin*)
*d*_v_	vlg-size-distribution	[-]	type of distribution for village size (*constant, uniform, normal, log-normal*)
*μ*_v_	vlg-size-mean_ha	hhs	mean of village size distribution
*σ*_v_	vlg-size-sd_ha	hhs	standard deviation of village size distribution
*d*_v_	vlg-min-distance	cell	minimum distance between villages
*d*_h_	hh-area-distribution	[-]	type of distribution for household area (*normal, log-normal*)
*μ*_h_	hh-area-mean_ha	ha	mean of household area distribution
*σ*_h_	hh-area-sd_ha	ha	standard deviation of household area distribution
*n*_i_	inaccessible-area-fraction	[-]	fraction of landscape covered by inaccessible area (e.g. large-scale plantations, protected area)
*l*_i_	inaccessible-area-location	[-]	location of inaccessible areas (either *random* or *road-connected*)
*d*_i_	inaccessible-area-distribution	[-]	type of distribution for inaccessible area (*constant, uniform, normal*)
*μ*_i_	inaccessible-area-mean	ha	mean of inaccessible area distribution
*σ*_i_	inaccessible-area-sd	ha	standard deviation of inaccessible area distribution
*t*_f_	field-type	[-]	*distribution*: field sizes are drawn from the provided distribution; *percentage*: mean of the field size distribution is adjusted to the percentage set by *μ*_h_
*d*_f_	field-size-distribution	[-]	type of distribution for field sizes (*constant, uniform, normal, log-normal*)
*μ*_f_	field_size_mean_ha	ha	mean of field size distribution
*σ*_f_	field_size_sd_ha	ha	standard deviation of field size distribution
*p*_f_	field-size-percentage	[-]	sets percentage to adjust *μ*_f_ by multiplication with *μ*_h_ (only used when *t*_f_ is *percentage*)
*s*_f_	field-shape-factor	[-]	controls if fields are mostly rectangular (value 1) or narrow (higher values)
*t*_strat_	strategies-type	[-]	type of field strategies selection; *manual*: choose establishment strategies by manual selecting; *id*: use a predefined list of strategies
*s*1_strat_	s1.homebase	[-]	field establishment strategy 1: establishment close to own home-base (true/false)
*s*2_strat_	s2.fields	[-]	field establishment strategy 2: establishment close to own fields (true/false)
*s*3_strat_	s3.nearby	[-]	field establishment strategy 3: establishment in nearby ‘others’ cell (true/false)
*s*4_strat_	s4.avoid	[-]	field establishment strategy 4: establishment in nearby ‘others’ cell surrounded by ‘others’ cells (true/false)
*n*_strat_	change-strategy	[-]	number of unsuccessful tries for field establishment after which search strategy is changed
*i*_strat_	field-strategies-id	[-]	overwrites manual strategies selection and determines a pre-specified list of search strategies for field establishment (e.g. s1, s2, s4)
*t*_1_	land-use-assignment	[-]	crop type assignment algorithm; *landscape-level-fraction*: approximate fractions of crop types; *landscape-level-specialization*: approximate fractions of crop types and fractions of household specialization
*id*_1_	LUT-l-name (l = 1,2,3,4,5)	[-]	name of crop types *l* (*l*=1,2,3,4,5)
*fr*_1_	LUT-l-fraction (l = 1,2,3,4,5)	[-]	fraction of agricultural area under crop type *l* (*l*=1,2,3,4,5); fractions must sum up to 1
*fr*_1,spec_	LUT-l-specialize (l = 1,2,3,4,5)	[-]	minimum fraction of area under crop type *l* (*l*=1,2,3,4,5), which is farmed by specialist households
*f*_1_	LUT-fill-up	[-]	crop type (ID) to fill up fractions if sum of *fr*_1_ is smaller than one

The initialization procedure first updates world dimensions according to the parameters *w*_*s*_ and *h*_*s*_. All cells in the landscape are initialized as ‘others’ cells, with no household or fields. At that stage, cells are not owned by anybody. Global variables are set according to the user inputs and the output plots of the model are refreshed. Next, a sample of the household size distribution is drawn to initialize household properties. While the creation and placement of household agents is performed by the submodel “Road creation and Household placement” (see Section Submodels), the properties of the household agents are already determined during initialization. EFForTS-LGraf provides three options for household initialization via the parameter *setup-type* (*t*_*s*_). Depending on the chosen *setup-type*, the user provides either (1) a fixed number of households *n*_s,h_ (option: households). The number of villages and the proportion of agricultural area are then approximated by using the defined village size and household size distributions; (2) the number of villages *n*_s,v_ (option: villages). The number of households and the proportion of agricultural area are then approximated by using the defined household size distribution; (3) the proportion of agricultural area *n*_s,a_ (option: area). The number of households and the number of villages are then approximated by using the defined household size and village size distributions.

Each of the above options generates a preliminary list of households. Each household has three properties: household-ID, household size and village-ID. The household sizes approximate the defined household size distribution, whereas the village-IDs are assigned in such a way that resulting village sizes approximate the defined village size distribution.

#### Submodels

Road creation and household placement. After initialization, first all roads are created and then households are placed along the established road network. Roads are treated as landscape items without a dimension, i.e. like polylines in GIS. Just as any other cell, cells with an intersecting road have a land-use type. There are three options for road creation: (1) a road network is created based on an existing road map in an input file (option: real.shapefile); (2) a road network is artificially created based on a random elevation model (option: artificial.perlin); or (3) a road network is artificially created based on the straight road creation algorithm of the G-Raffe landscape generator (option: artificial.graffe). For details on road creation and household placement see section 1.1.1 in S1 File.

Once the road network is established, households are created and placed along the road network. First, the algorithm determines the number of villages depending on the pre-generated list of households from the initialization procedure. Then, based on the number of villages and village-IDs, village centers are created on random road network cells complying with the minimum distance between village centers, *d*_v_. Then the households are placed randomly on road cells around village centers matching the corresponding village-IDs, i.e. each household establishes a home-base cell at the assigned cell. There is a cap to the number of household home-bases on one cell *n*_s,c_.

Inaccessible areas are an optional landscape feature of EFForTS-LGraf, defining patches of areas that are not available for use by smallholder agriculture. This option allows defining either areas belonging to large-scale company plantations or protected (forest) areas. Given the overall fraction of the landscape covered by inaccessible area, *frac*_*p*_ (Table 1) and the inaccessible area size distribution, patch sizes are drawn from the distribution until the total size of inaccessible area patches matches the defined landscape fraction. From this list, each patch is then created by first selecting a starting location, which can be either a random cell in the landscape (option *random* for *l*_*i*_) or a random road cell (option *road-connected* for *l*_*i*_). From this starting location, a square-shaped field of the given size is created, following the basic field establishment rules (details see section 1.1.2 in S1 File).

Field establishment. The field establishment procedure determines the size and spatial location of fields, but does not yet determine the actual crop within fields. This is assigned in the next procedure (see “Land-use assignment” below). Fields are established by household agents and an attempt to establish a field comprises three steps: (1) deciding on the field size, (2) moving to a potential location, and (3) making sure there is enough space to establish a field of the desired size in this location (for details see section 1.1.2 in S1 File). In case of a successful attempt, the household gains ownership of these established field cells. By gaining ownership, the realized household area increases and field establishment continues until each household realizes its expected household size that was determined during initialization. In other words, to realize the expected household size distribution, the procedure loops over all households that are marked as still growing, i.e. all households where the realized area of owned fields does not yet exceed the expected household size. At the first stage, these are all households because each household establishes at least one field. In every iteration, each relevant household draws a field size from the field-size distribution. If the projected household area, including the additional field, is below the expected household size, then the household attempts to establish a field of this size. If the projected area exceeds the expected household size, an attempt for establishment would take place only if the absolute difference between projected household area and expected household area is smaller after establishing the field. Otherwise, the household does not establish the field and becomes a non-growing household. After each establishment loop, households that have reached their final size become non-growing households, namely are excluded from the next iteration of field establishment. At the end of each loop, if the total realized agricultural area exceeds the expected cover of agricultural area, the field establishment procedure is halted.

Crop type assignment. After all fields are established, crop types are assigned to them. The current model version supports up to five different crop types and two alternative ways to distribute the crop types (parameter *t*_*l*_). The first option (*landscape-level-fraction*) distributes crop types randomly among fields according to fractions of overall crop types. The second option (*household-level-specialization*) aims at additionally incorporating specialization for crop types at the household level. In this latter case, an additional input parameter is used for each crop type which describes the specialization by households for this particular crop type. The level of specialization is primarily a proportion ranging from 1 (all households that cultivate this crop type would cultivate this crop type exclusively) to zero (no preference for specialization in this crop type). For example, a specialization value of 0.7 for crop type 1 would mean that 70% of households that cultivate crop type 1 have only fields of crop type 1 and no field of any other crop type. The remaining 30% of households have fields of various crop types. Note that realized specialization levels can be higher than the input specialization levels, since the value determines a field-level outcome, and hence, all households with only one field are specialists by default.

#### Output data

The landscapes produced by EFForTS-LGraf contain information on various spatial scales (landscape level, household level, fields, cells) and can be visually inspected in different formats:

- Land-use maps: depict land uses in different colors (classes: agriculture, road, inaccessible, home-base, ‘others’)
- Crop type maps: similar to land-use map with additional classification of crop types
- Agriculture-non-agriculture map: depicts the distinction between agricultural cells and cells of class ‘others’
- Field-patches map: depicts the different fields on the map in different colors
- Household-patches map: differentiates fields based on the different households they belong to
- Habitat-patches map: depicts clusters of cells belonging to the same patch of class ‘others’ in different colors. Roads, fields and inaccessible areas function as separators for patches.

Maps are produced at the resolution cell-length-meter *c*_s_. Cell labels such as patch-IDs and owner-IDs, and spatial elements such as roads, home-bases and households can be selected to be drawn on top of these maps. All spatial outputs can also be stored as raster maps (ASCII) to allow using the generated landscapes for other model applications (Details on raster output see section 1.1.3 in S1 File). An additional feature (“create-3D-map” function) uses the NetLogo 3D functionality to create a 3D rendered map that displays trees, crops and buildings using 3D-shapes in realistic densities (Fig A1 in S1 File).

## Scenarios and parameterization

**Fig 3:**
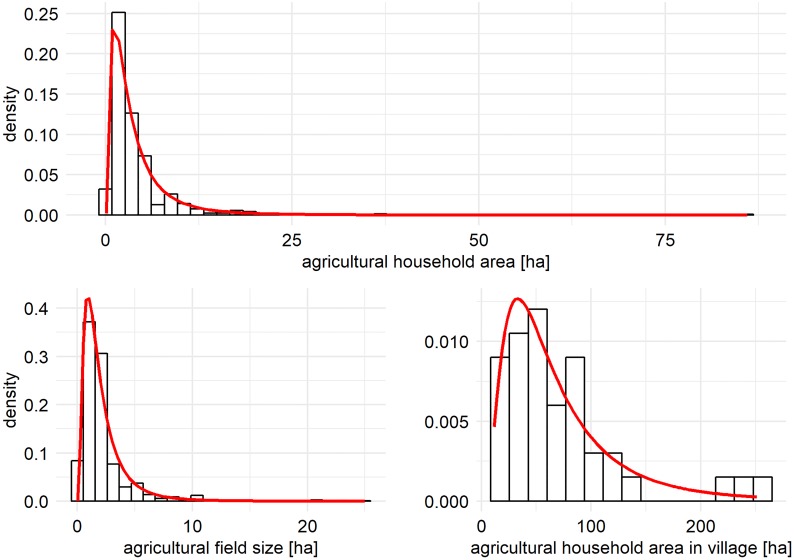
Distribution of household areas, village areas and field sizes, based on household surveys carried out in our study area in Jambi province, Sumatra, Indonesia.

We demonstrate the capabilities and potential uses of EFForTS-LGraf based on the example of smallholder-dominated agricultural areas in Jambi province in Sumatra, Indonesia. During the last decades, this region has faced severe land-use changes, mainly deforestation and agricultural expansion (e.g. [23]) and loss of ecosystem-functioning of the transformed landscapes (e.g., [24]). In order to provide agricultural maps that incorporate smallholder households and field ownership, we parameterized EFForTS-LGraf using household data from a smallholder survey of relatively large size (701 farming households) that was performed in Jambi province (Fig 3) [25, 26]. Jambi province is characterized by small villages with farming households that are mostly of relatively small size (median survey data 3.5 ha) and within these villages, small-scale fields with mostly oil palm and rubber. By parameterizing EFForTS-LGraf for a specific study region, the generated maps can be used to inform scenario-based studies such as the application of the agent-based simulation model EFForTS-ABM [16].

To show EFForTS-LGraf’s model and output capabilities, we followed three approaches. In approach 1 (Sobol sensitivity analysis), we determined how variability in the landscapes generated by EFForTS-LGraf can be apportioned to the different model parameters. In approach 2 (Validation), we assessed the characteristics of typical landscapes in the Harapan region in Jambi province and applied an optimization algorithm in order to generate artificial landscapes with similar characteristics. In approach 3 (Applied case study), we present an applied simulation scenario that investigates effects of household consolidation and crop specialization on landscape characteristics. For all approaches, we quantified the landscape characteristics using five landscape metrics that are either class-based or aggregated on class-level: landscape-shape-index (LSI), largest-patch-index (LPI), mean-patch-area, the number of patches (n-patches) and patch-cohesion-index (PCI) (Table 2, for a more detailed description see FRAGSTATS manual [27]). We used the R-package *SDMTools v1.1* to calculate landscape metrics of all landscapes [28].

**Table 2:** Landscape metrics description.

landscape metric	short	range	description
landscape-shape-index	LSI	*LSI* ≥ 1, *without* *limit*	measure of class aggregation or clumping
largest-patch-index	LPI	0 < *LPI* ≤ 100	percentage of total landscape area comprised by the largest patch of a class
mean-patch-area	-	≥0, *without* *limit*	mean patch area of all patches of a class
n-patches	-	≥0, *without* *limit*	total number of patches of a class
patch-cohesion-index	PCI	0 ≤ *PCI* ≤ 100	physical connectedness of patches of a class

For approach 1, we conducted a Sobol sensitivity analysis, i.e. a global variance-based sensitivity analysis for all model parameters including the parameters of the artificial road creation algorithm of EFForTS-LGraf (for parameterization details, see section 1.2.1 in S1 File). The Sobol method measures direct effects and interaction effects of model parameters on model output (here: landscape metrics) [29–32]. By applying a Sobol parameter sampling design, we generated 9500 different landscapes that cover a large parameter range of EFForTS-LGraf. Such analysis helps understand model processes and may be useful to reproduce certain landscape features. For instance, if one is interested in generating landscapes along a gradient of characteristics, the sensitivity analysis allows identifying which parameters can realize that gradient and how.

**Fig 4:**
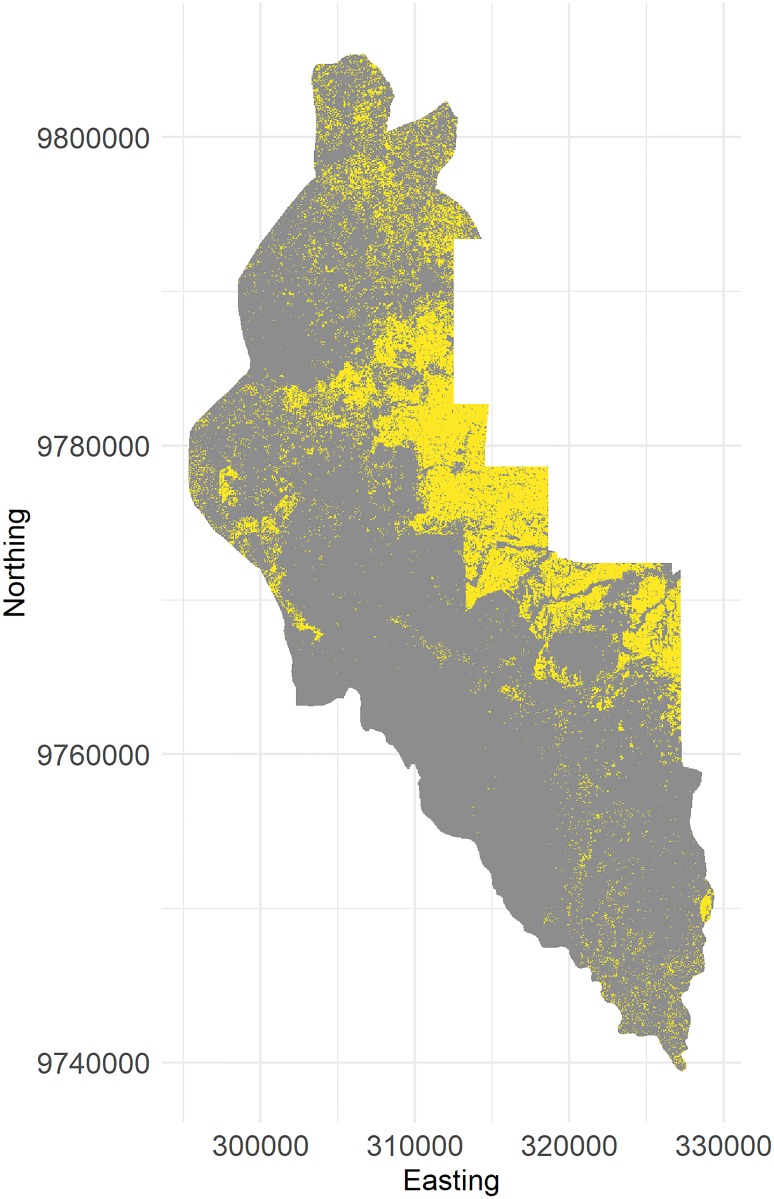
Snapshot of the reclassified satellite image of Harapan region in Jambi province. Grey cells indicate land-cover type ‘others’, which consist mostly of secondary forest but includes all other remaining non-agricultural land-cover classes, such as settlements and water bodies. Yellow cells indicate fields, which consist of oil palm and rubber plantations.

For approach 2 (Validation), we used a land-cover map (classified satellite image from 2013) from the Harapan region in Jambi province that features a large gradient of land-use intensities [33] (see Fig 4). The original land-cover map has a spatial resolution of 5 × 5 m and an overall classification accuracy of 68.4% [33]. In order to allow for comparisons with our artificially-generated landscapes, we scaled the land-cover map to the same resolution as our generated EFForTS-LGraf landscapes, which is 50 × 50 m cells. The original classified land-cover map consisted of 9 classes which we reclassified into two final land-cover types: fields and ‘others’ (fields consist of original classes rubber and oil palm; ‘others’ consist of original classes secondary dryland forest, shrub, bare land, settlement, water body, cloud, shadow). While we did not explicitly assess the overall classification accuracy of the final reclassified and rescaled map, overall classification accuracy is expected to improve trough reclassification into more general classes. From the reclassified land-cover map, we sampled 3 randomly placed landscapes, 100 × 100 cells in size (no overlaps) and calculated the five landscape metrics for each of these sampled landscapes and each land-cover type (fields and other). For each sample, we performed a genetic algorithm in order to recreate these samples with EFForTS-LGraf [34, 35]. The algorithm uses the proportion of agricultural area from the samples, but varies all road, household, village and field establishment parameters. For each generated landscape, a fitness value is calculated by comparing the landscape metrics of the generated landscape to the landscape metrics of the current sample. The algorithm then tries to minimize the total deviance by repeated creation of landscapes with adjusted parameters. The genetic algorithm was set up with 50 different initial parameterizations per sample (population size) and 25 iterations. In approach 2 (validation), we also compared the ranges of landscape metrics of the 9500 generated landscape from the Sobol sensitivity analysis (approach 1) to landscape metrics of 100 landscapes. These landscapes were randomly sampled (allowing overlaps) from the reclassified land-use map (results, see section 1.2.2 in S1 File).

EFForTS-LGraf not only allows us to generate land-cover maps, but also to conduct applied modelling studies (approach 3). Empirical data from Jambi, Indonesia indicates an increase over time in the area owned by households [36]. Smallholder households also tend to specialize on one specific crop type [37]. Increasing household area and specialization on one crop may cause drastic changes in landscape composition and configuration. We analyzed whether EFForTS-LGraf can reproduce these changes by generating a set of landscapes with different levels of crop specialization and household area using a Latin hypercube sampling design with 500 samples [38]. We randomly selected one village from the household survey in Jambi and cropped a road polyline shapefile based on a spatial layer covering the road network of Jambi. We used the classified satellite image of the village to estimate the proportions of oil palm and rubber in the agricultural landscape (*oilpalm* = 0.5, *rubber* = 0.5). To mimic the increase in household area, we set the proportion of agricultural area in the landscape to a fixed value of 50% and varied the mean of the log-normal household area distribution from 1 ha to 3 ha within the Latin hypercube sampling design. The total number of households in the landscape was then estimated based on the proportion of agricultural land. With increasing mean values of the household area distribution, this yielded fewer households but the same total agricultural area in the landscape. We used two crop types (*oil-palm* and *rubber*) and varied the specialization levels for *oil-palm* from 0 (specialist by chance) to 1 (always specialists) within the Latin hypercube sampling design. For crop type 2 *rubber* we set the specialization level to 0 (for parameterization details, see section 1.2.3 in S1 File). We analyzed the resulting landscapes via the five selected landscape metrics from previous approaches (Table 2). We calculated linear regression models for each landscape metric and crop type combination and calculated standardized regression coefficients to estimate parameter and interaction effects on landscape metrics of the generated landscapes.

Execution of NetLogo simulations and output post-processing where performed with R and the R-package nlrx [39, 40].

## Results

### Approach 1: Sobol sensitivity analysis

**Fig 5:**
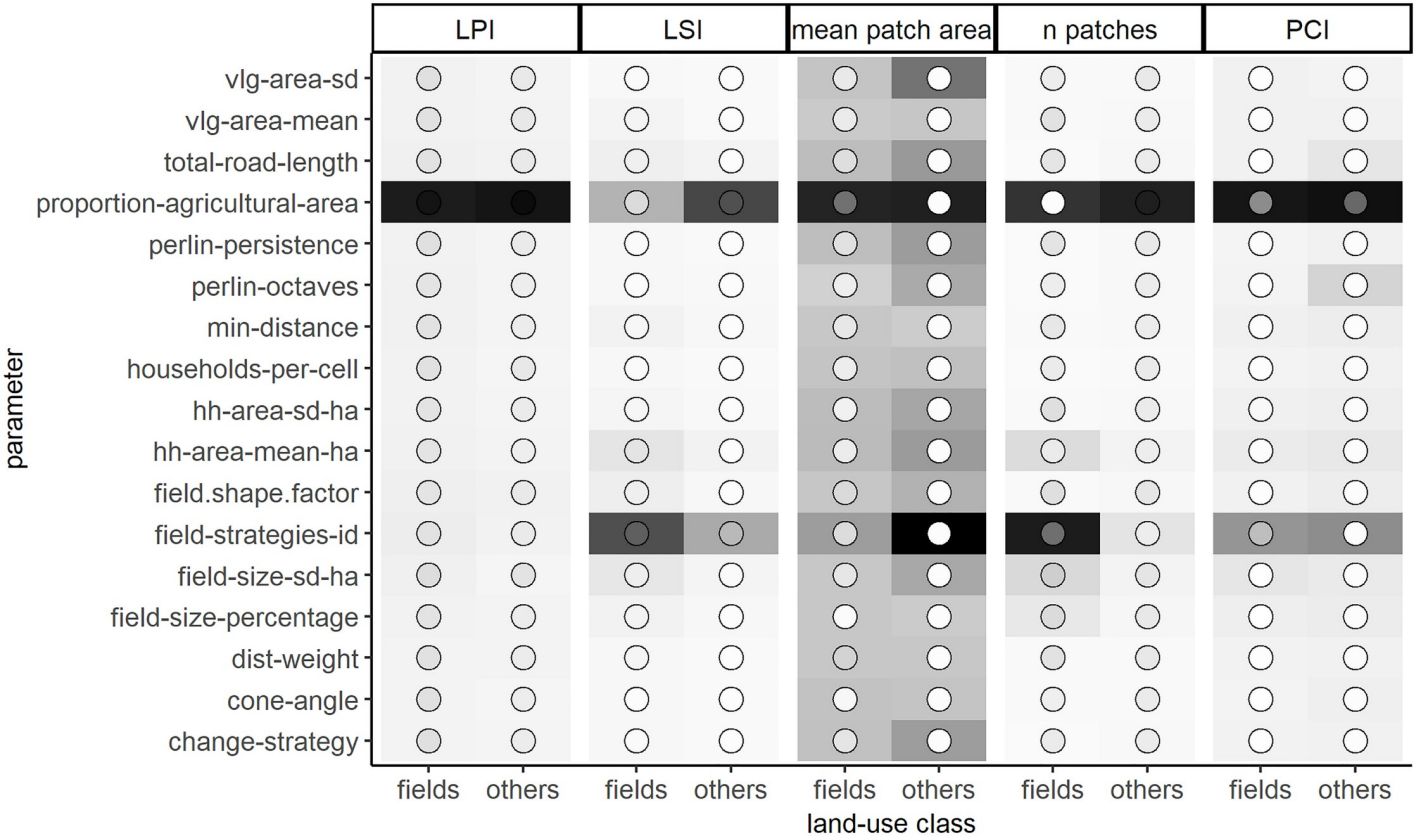
Approach 1, sensitivity analysis: Sobol total and main effects of EFForTS-LGraf model parameters on landscape metrics grouped by land-use classes fields and ‘others’. Tile color of each parameter output combination indicates the total effect of parameter changes on the output metric. Colors of dots within each tile show the main effect of parameter changes on the output metric. Thus, tiles with dark color and a bright dot have a large total effect but a small main effect indicating strong interaction effects, whereas tiles with dark color and a dark dot indicate strong main effects. For abbreviations and model parameterizations, see section 1.2.1 in S1 File.

Two parameters, *total-agricultural-area*, defining the resulting proportion of agricultural area in the generated landscapes and *field-strategies-id*, defining the set of field establishment strategies that is used by the households, had significant total (sum of direct and interaction effects) and main effects (direct effects without interaction) on a wide range of landscape metrics (Fig 5). For some landscape metrics such as LPI or LSI these two parameters showed only main effects (indicated by dark tile and dot shading in Fig 5). The largest patch index (LPI) was only affected by the parameter *proportion-agricultural-area*. The mean patch area was the only output landscape metric that was significantly affected by all model parameters. For some output metrics (LSI, n patches, PCI), the *proportion-agricultural-area* had a larger effect on ‘others’ class indices, whereas the *field-strategies-id* had a larger effect on the fields indices. Interestingly this pattern was reversed for the output metric mean patch area, where the *proportion-agricultural-area* had a larger effect on the mean patch area of field patches and the *field-strategies-id* had a larger effect on the mean patch area of ‘others’ class patches.

### Approach 2: Validation

The re-classified satellite image of the Harapan region, Jambi, shows a large heterogeneity in the distribution of agricultural patches (Fig 4). Large-scale clustered agricultural areas can be found in the North-East, whereas in the North, West and South, agricultural patches are more scattered. Harapan rainforest conservation area is located in the centre of the image and does not contain any agricultural fields at all.

**Fig 6:**
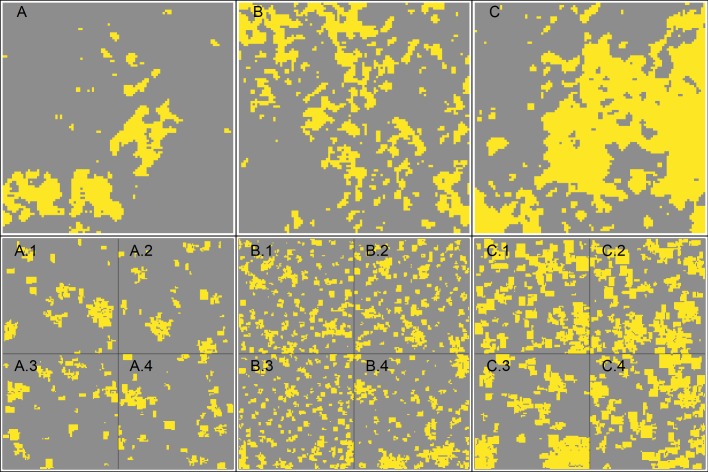
Approach 2, validation: A, B and C show sampled maps (100 × 100 cells, 50 m resolution) from the reclassified satellite image of the Harapan region, Jambi province, Indonesia. Yellow cells indicate agricultural area, grey cells indicate land-use class ‘others’. We applied genetic algorithm optimization to tweak EFForTS-LGraf model parameters in order to recreate these map samples. This was done by calculating deviances in landscape metrics between the sampled map and the generated map and minimizing this deviance with each generation of the algorithm. We ran the algorithm for each map sample (A, B, C) individually and stored the final parameterization with the lowest deviation. Using these final parameterizations we generated 4 maps for each map sample to account for stochasticity during the map creation process (A.1-A.4, B.1-B.4, C.1-C.4). The generated maps have the same resolution as the map samples (100 × 100 cells, 50 m resolution) but are displayed at 1/4th size.

**Fig 7:**
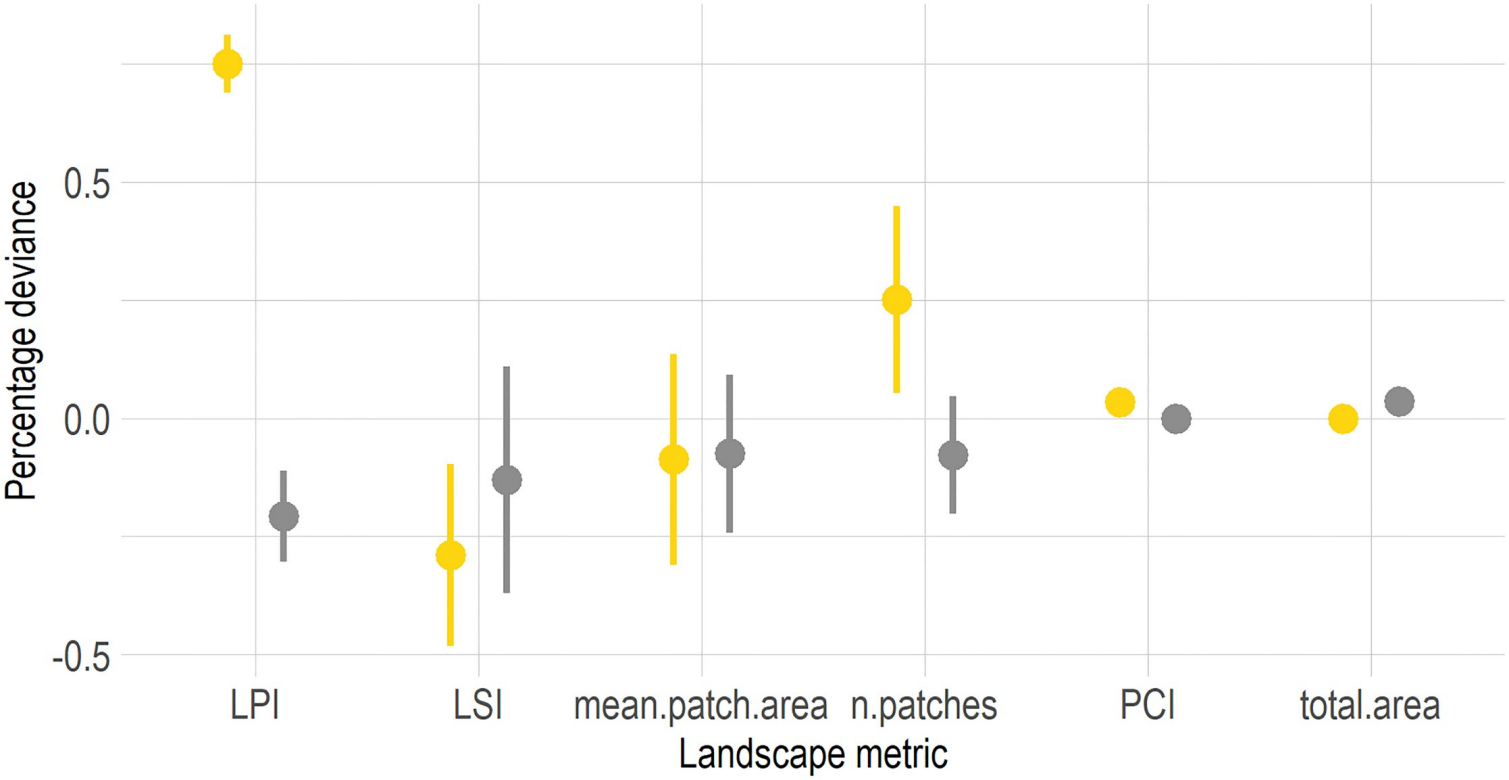
Approach 2, validation: Landscape metrics deviations of generated maps derived by application of a genetic algorithm (see maps A.1-A.4, B.1-B.4, C.1-C.4 in Fig 6), to landscape metrics of original samples from the reclassified satellite image of the Harapan region, Jambi province (see maps A,B,C in Fig 6). Yellow dots and line ranges represent landscape metrics of agricultural patches, grey dots and line ranges those of patches of class ‘others’.

The three landscapes that were sampled from this map reflect this gradient with agricultural proportions ranging from of 0.13 (sample 1, Fig 6) over 0.23 (sample 2, Fig 6) to 0.46 (sample 3, Fig 6). All three samples also show a large heterogeneity in patch sizes, field sizes and distribution of fields (see Fig 6). While the genetic algorithm was able to find parameterizations that recreate many properties of the sampled landscapes, not every detail could be matched (see Fig 7). Especially large patches of agricultural area could not be recreated accurately (yellow ranges for largest patch index (LPI) and n.patches, Fig 7). However, even the highest deviation (LPI of agricultural patches) was still below 1%.

### Approach 3: Applied case study

**Fig 8:**
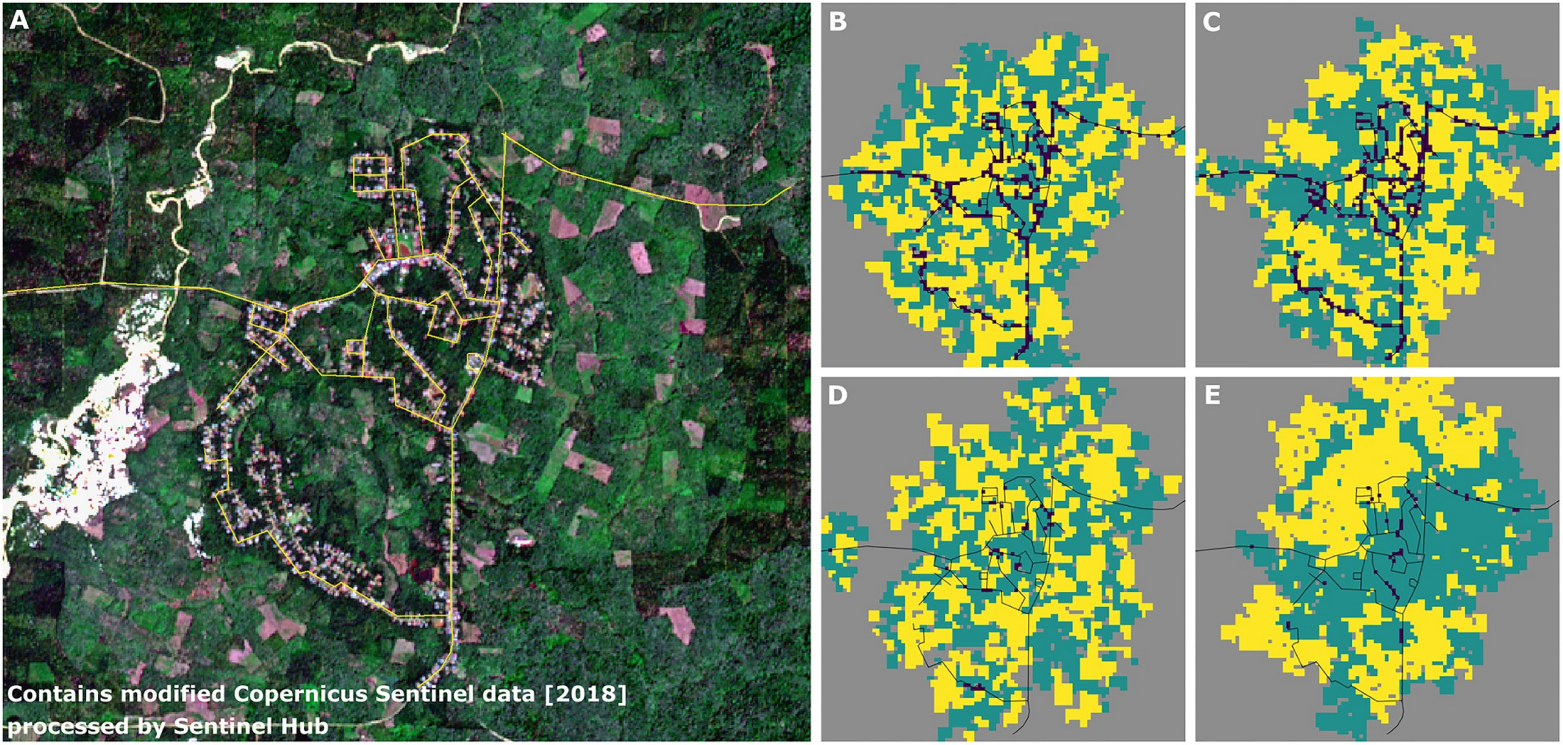
Approach 3, applied case study: (A) Satellite imagery showing the village Lantak Seribu in Renah Pamenang District, Merangin Regency, Jambi (contains modified Copernicus Sentinel data [2018] processed by Sentinel Hub). The road network of this village (yellow lines) was selected to generate examples of artificial agricultural smallholder landscape maps with EFForTS-LGraf for different household sizes and specialization levels. (B-E) Examples of artificial land-cover maps. Green cells indicate oil palm fields, yellow cells indicate rubber fields, grey cells indicate cells of class ‘others’, purple cells indicate household home-bases and black lines indicate roads. Examples B and C consist of smaller households that own only some fields whereas households in D and E are larger and own more fields. In B and D, land uses are distributed to fields completely at random, whereas in C and E, households specialize completely on one land use.

In all generated landscapes, fields were distributed mainly along the road network that was used to set up the model (Fig 8). We observed inter-linking effects of household area and specialization on aggregation of crop types in the landscape by visual comparison of resulting land-use maps (Fig 8). High specialization on oil palm led to much higher spatial aggregation of crop types when household area was larger, compared to smaller household area (Fig 8).

**Fig 9:**
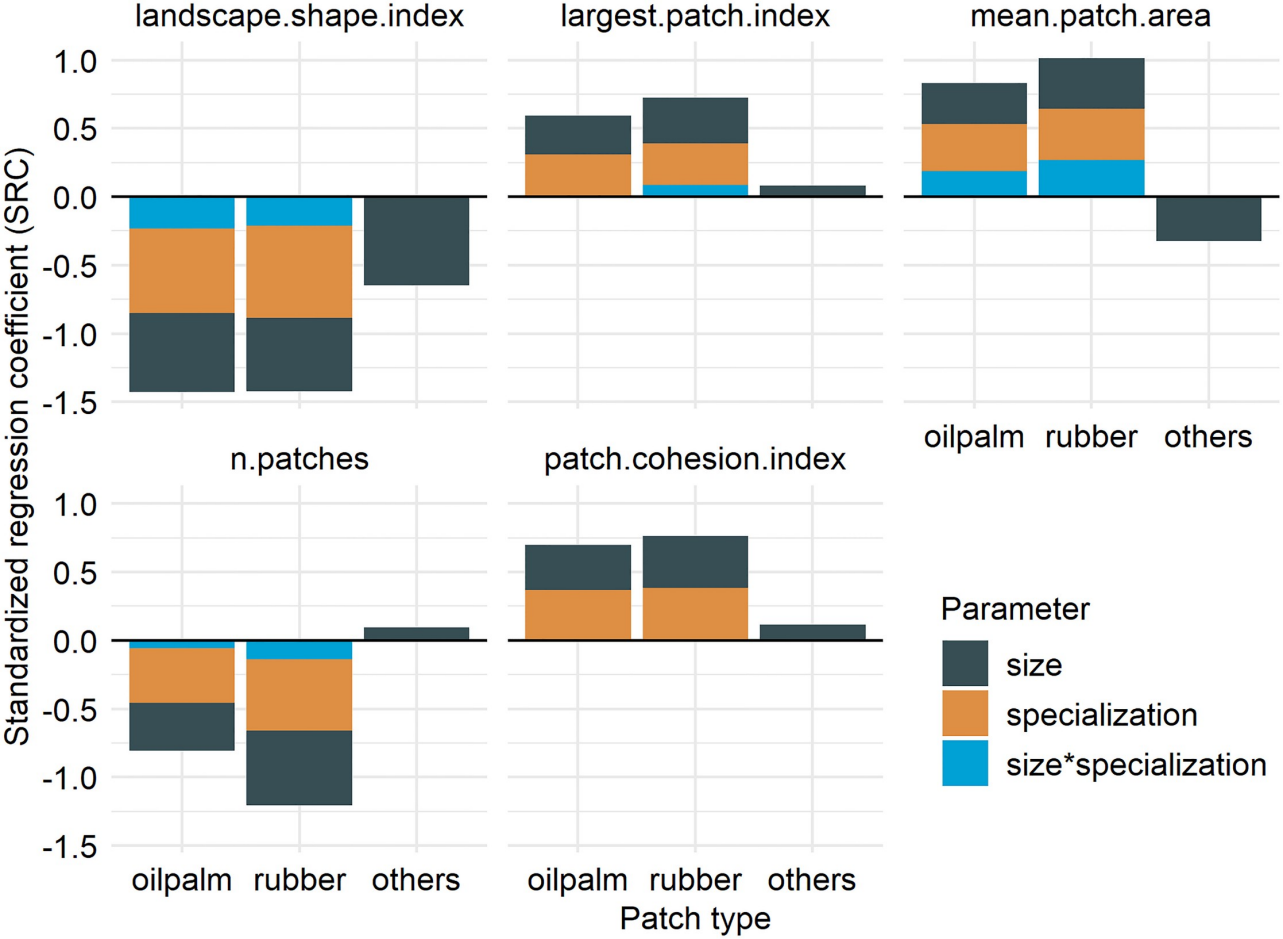
Approach 3, applied case study: We generated land-cover maps with varying household area and specialization levels for oil palm and calculated five selected landscape metrics for the two crop types (oil palm and rubber) and patches of class ‘others’. The colored bars illustrate the corresponding standardized regression coefficients (SRC) from linear model regressions. Bars display significant importances of household size (size), specialization level (specialization) and the importance of interactions between these two parameters on the selected landscape metrics. Parameter names and values are described in section 1.2.3 in S1 File. Landscape metrics are described in Section Scenarios and parameterization, Table 2.

Within the boundaries of our assumptions we did not find any significant effects of oil palm specialization (specialization, orange bars in Fig 9) or interaction effects (size*specialization, blue bars in Fig 9) on ‘others’ landscape metrics (see standardized regression coefficients for ‘others’ patches in Fig 9). However, all metrics were significantly affected by the household size distributions (size, black bars in Fig 9). For increasing household areas we found increased aggregation (negative landscape.shape.index coefficient), smaller patches (negative mean.patch.area coefficient), slightly higher total number of patches (positive n.patches coefficient) and fewer perimeter cells (positive patch.cohesion.index coefficient) of ‘others’ patches (Fig 9).

The landscape metrics of agricultural patches of the two crop types where clearly affected by household area, specialization on oil palm and interaction effects of both parameters (see oil palm and rubber bars in Fig 9). In a system with two mono-cultural crop types, high specialization on one crop type indirectly affects the spatial distribution of the other crop type. Patch aggregation was higher for both crop types with increasing specialization and household area (negative landscape.shape.index coefficients). Mean patch area was higher for high specialization and larger households (positive mean.patch.area coefficients). Accordingly, the number of rubber and oil palm patches strongly decreased with increasing household area and oil palm specialization (negative n.patches coefficients). There were fewer perimeter cells for high specialization levels and larger household areas (positive patch.cohesion.index coefficients). Landscape shape index and mean patch area showed a considerable amount of interaction effects between both parameters suggesting non-linear relationships, which could also be observed from investigating raw data from the simulations (see Fig A4 in section 1.2.3 in S1 File).

## Discussion

Although various landscape generator approaches have been developed in the past, only few of them are process-based, have a distinct focus on agricultural land or incorporate any human dimension [7]. The main goal of our study was to fill this gap by developing the landscape generator EFForTS-LGraf. The process-based algorithms of EFForTS-LGraf explicitly reflect the linkage of agricultural expansion and deforestation to road and infrastructure development [17–19]. This allows not only to generate realistic-looking landscapes, but also links the model to one of the world’s central sources of environmental pressures, i.e. road expansion [12]. Another key power of the model is explicit consideration of smallholder farming households and elements that characterize smallholder decisions and socio-economic patterns such as land ownership and farm economy—and how these shape landscapes. In this, the model helps overcoming an important barrier between ecological and socio-economic research, allowing one to explore the behavior of agricultural-natural frontiers as a socio-ecological system [41].

The process-based nature of the model algorithms and parameters allow to formulate and evaluate specific questions through adjustment of the model parameterization. Particularly the final analysis (approach 3) demonstrates how the model can be used to explore how factors that affect smallholder decisions and socio-economic processes shape landscape patterns and, thereby, biodiversity and ecosystem services. Notably, global processes of agricultural intensification comprise two elements that are captured by the model, namely a trend of specialization toward monocultures and an increase in field sizes and area owned by fewer smallholders [42], accompanied by an ongoing decline in rural employment (e.g. [43] for the EU). Our simulation experiment revealed that household consolidation and crop specialization indeed had a large impact on various landscape characteristics. Although these effects were expected to some extent, our analysis showed that the inter-linkages between household-level processes and landscape characteristics were significantly affected by interaction effects of consolidation and specialization. Such identification of interactions and non-linear relationships may be important for a wide range of ecological studies.

We assessed the basic functionality, parameter sensitivity and validity of EFForTS-LGraf by performing a sensitivity analysis and a validation against real-world satellite imagery. The Sobol sensitivity analysis (approach 1) revealed that the proportion of agricultural area (*proportion-agricultural-area*) and the selection of establishment strategies (*field-strategies-id*) were the most important parameters across all landscape metrics. However, the effect of each parameter highly depended on the chosen landscape metric. For example, mean patch area was affected by nearly all model parameters, whereas the largest patch index was nearly exclusively affected by the proportion of agricultural area. We used the *setup-type ‘area’* to initialize the households of each model simulation. Therefore, household area parameters by definition had little influence, since they were largely pre-set. This also means that we can expect larger household area parameter effects on landscape metrics when using the *setup-type ‘household’*. The three different *setup-types* of EFForTS-LGraf allow for great flexibility in parameterizing the model. Depending on the application, it may be important to generate landscapes with the same proportion of agricultural area, but different household properties (approach 3). When the total number of households in the area is unknown but the typical village sizes and the number of villages in the area can be estimated, the *setup-type village* can be used to approximate the number of households.

Our artificially-generated landscapes showed high capacity to match various landscape metrics when compared to classified satellite images, and showed high flexibility to generate a broad range of maps along a gradient of spatial structures (see Figs 4 and 7 and section 1.2.2 in S1 File). Both are important features of landscape generators. Depending on the approach, it may be important to recreate specific maps trough pattern-based optimization approaches or to generate many different maps along a gradient of specific landscape characteristics.

Besides these technical approaches, EFForTS-LGraf can be applied for a wide range of potential applications. First of all, the generated maps can be used to inform other modelling studies, as has been successfully done with the simulation model EFForTS-ABM [16]. We are also planning to apply and validate EFForTS-LGraf to other agricultural regions where high quality remote sensing data are available, such as Central Europe. Additionally, EFForTS-LGraf may be required to perform policy-relevant applications, e.g., testing the future impacts of road expansion, especially in developing regions such as Indonesia and impacts of agricultural policies such as the CAP in the EU.

Currently, EFForTS-LGraf produces maps for one specific point in time. Considering the huge pressures of road expansion on natural habitats, particularly in developing countries including in Indonesia [13], incorporating a temporal component explicitly might be a useful extension to the model. Such an extension would allow to create time series of maps with roads and fields occurring gradually. The assumption that at the beginning of map generation, each cell is equally suitable for field establishment is another limitation of EFForTS-LGraf. A potential model extension could add heterogeneous land-use types to the initial state of the landscape (e.g. forest, grassland, peatland, instead of assigning type ‘others’ to all cells). By consideration of differential pressure for land-use change depending on these land-use types, more complex landscape patterns could be created. We decided to develop EFForTS-LGraf with a clear focus on linking geospatial information, such as road polyline shapefiles and land-use fractions, with empirical data, such as household size and field size distributions.

When comparing model outcomes with reclassified land-cover maps from Harapan region (approach 2), we also revealed some systematic differences. Most prominently, the area of agricultural patches was underestimated in the generated landscapes, whereas the area of ‘others’ patches was overestimated. This mismatch may partly be explained by the homogeneous field establishment of model households. Although households are able to adjust their field establishment strategies if they are not successful, each household uses the same set of strategies and switches after the same number of unsuccessful tries. Adding household-level heterogeneity to field establishment strategies would allow for increased local field aggregation heterogeneity but would also add complexity to the model. In the current model version, crop types can be assigned to fields based on the user-set fractions and specialization levels. Future model extensions may also contain additional algorithms to control spatial clustering of certain crop types.

In conclusion, EFForTS-LGraf combines economic smallholder survey data and spatial information to generate landscapes featuring the characteristics of observed agricultural smallholder landscapes. EFForTS-LGraf is especially useful for applications where agricultural maps need to be provided in conjunction with corresponding economic household data which can not be obtained from remote sensing alone. Due to its flexibility, EFForTS-LGraf can be utilized for a wide range of applications, such as: (1) map generation by providing specific economic case study data, (2) application of pattern-matching approaches to match generated maps with realistic land-use maps, and (3) generating maps along gradients of properties on household or landscape-level. EFForTS-LGraf contributes to the set of already published landscape generators and fills an important gap through its application of process-based algorithms with a distinct focus on road expansion, agricultural land and explicit consideration of human dimensions of land-use change.

## Supporting information

S1 File Appendix: EFForTS-LGraf model and analysis details. (PDF)
