# Supplementary material for: EFForTS-LGraf: A landscape generator for creating smallholder-driven land-use mosaics
Source: PLoS One. 2019 Sep 27;14(9):e0222949. doi: 10.1371/journal.pone.0222949 (PMC6764663; doi:10.1371/journal.pone.0222949)
Supplement: S1 File — (PDF) [file pone.0222949.s001.pdf]

EFForTS-LGraf: A Landscape Generator for Creating Smallholder-Driven Land-Use Mosaics

Jan Salecker[1,*], Claudia Dislich[1¤], Kerstin Wiegand[1,2], Katrin M. Meyer[1], Guy Pe´er[3,4,5]

1 Ecosystem Modelling, Faculty of Forest Sciences and Forest Ecology, University of Goettingen, Goettingen, Germany
2 Centre of Biodiversity and Sustainable Land Use (CBL), University of Goettingen, Goettingen, Germany
3 Synthesis centre (sDiv) of the German Centre for Integrative Biodiversity Research (iDiv) Halle-Jena-Leipzig, Leipzig, Germany
4 UFZ - Helmholtz Centre for Environmental Research, Dept. Economics and Dept. Ecosystem Services, Leipzig, Germany
5 University of Leipzig, Leipzig, Germany

¤Current Address: Graduate School HIGRADE, Helmholtz Centre for Environmental Research - UFZ, Leipzig, Germany
* corresponding author: jsaleck@gwdg.de

1 Appendix

1.1 Model Details

1.1.1 Details on road creation and household placement

EFForTS-LGraf offers three options for road creation: (1) roads can be read in from an existing road map (option: real.shapefile); (2) a road network is artificially created based on a random elevation model (option: artificial.perlin); (3) a road network is created based on the straight road creation algorithm of the G-Raffe landscape generator (option: artificial.graffe).

To upload an existing **real road map**, one needs to store the respective shapefile of the roads, the projection file and a second shapefile that only includes the extent of the map. After loading the shapefile, the world is re-sized if necessary. All cells that have a road intersecting are identified as road cells.

The **Perlin** algorithm mimics situations in which roads are created depending on elevation gradients. First, a simple perlin noise elevation model is created by adding several random noise grids with decreasing weight (perlin parameters $p1_{\mathrm{r,perl}}$, $p2_{\mathrm{r,perl}}$, $p3_{\mathrm{r,perl}}$) [1, 2]. The first road is then created by connecting two random locations in the landscape. First, a road-building-agent is created on one of these two random locations. For each neighboring cell, the road-building-agent calculates the distance to the destination cell (second random location) and the elevation difference to the agents' current location. The total score of each cell is then determined by weighting the distance and elevation criterion depending on the continuous parameter $p4_{\mathrm{r,perl}}$ (€[0,1]). The road-building-agent then moves to the cell with the highest score, establishes a road-cell there and continues to move and create road-cells until it reaches the final destination. Thus, by adjusting the parameter $p4_{\mathrm{r,perl}}$, the algorithm gives a higher weight to the distance (values near 1) of the connection allowing for straight roads or to the elevation gradient (values near 0) of the connection allowing for wiggling roads. If the $n_{\mathrm{r,art}}$ is not reached yet, another road is created by selecting one random point on an already established road and another location in the landscape that is at least $m_{\mathrm{r,art}}$ cells away from any other road.

Under the **G-RaFFe** algorithm all roads are straight and start along the left or lower edge of the simulation area and are directed either vertically, horizontally or diagonally within the landscape. Road length is drawn randomly within the interval $[1, \sqrt{w_s^2 + h_s^2}]$. The parameter $m_{\mathrm{r,art}}$ determines the minimum parallel distance in cells between two roads. Roads are created until the number of road cells reaches $n_{\mathrm{r,art}}$. This road creation option is in most parts adopted from the road creation algorithm of the G-RaFFe model [3].

1.1.2 Details on field establishment

During the EFForTS-LGraf field establishment procedure, households attempt to establish a field in three steps: first deciding on the field size, second moving to a potential location and third making sure there is enough space to establish a field of the desired size in this location.

Step one: The size f of the field is drawn randomly from the field size frequency distribution. Step two: Move to an 'others' start cell using one of four search strategies, which can change during the model run:

1. Random walk to an 'others' cell starting from the home base (*s1.homebase*)

2. Random walk to an 'others' cell starting from one of the fields of this household (*s2.field*)

3. Determine an 'others' cell within a defined radius from the home base (*s3.nearby*)

4. Determine the closest 'others' cell which is surrounded only by 'others' cells (*s4.avoid*)

The selection and order of these search strategies can be defined on the model interface. At the start cell, the household turns in a random compass direction (north, east, south or west) and starts with the field establishment procedure by trying to establish a first row of cells that will belong to the field. The household agent moves forward cell by cell until it either meets a cell which is already a field or a home base or an inaccessible area cell. To ensure reasonable field shapes, the minimum length l of this first row needs to be the side length of the smallest square that fits into the field size. The maximum length of this first row is then set to be the side length of the next larger square that fits into the field size. Because this algorithm restricts field sizes to rather quadratic shapes, the maximum length can be further modified by the parameter s_f which allows to proportionally increase the length of the first row. This results in narrower field shapes.

If the establishment of the first row is not successful, the other possible compass directions for the first row are tested. Once a first field row is established, the farmer tries to expand the field to the left or right of this row until the field reaches its predefined size f. If expansion to one side of the first row did not result in the designated field size, the field is extended also to the other side. If also this expansion does not yield the designated field size, all so far added field patches are removed, the household moves back to the start cell and starts over again with the first field row in a different direction. If all possible four compass directions do not lead to a successful field establishment, the farmer selects a new random start cell and tries again to establish a field of the designated field size f. The parameter n_{strat} defines in how many locations a household tries to establish fields before it switches the search strategy to the next strategy. If the household failed to establish fields for all selected strategies, the model will stop and report a warning message that field establishment was not successful. In such cases, the resulting landscape is incomplete because not all households reached their final size. This might especially happen under certain model parameterizations where households and field sizes tend to be very large without adjusting the total dimensions of the landscape.

1.1.3 Details on EFForTS-LGraf spatial output

The spatial information of generated landscapes can be exported as *.asc raster files in order to allow exchange to other model applications. EFForTS-LGraf contains export functions for the following raster files:

- Road-raster: 0 for non-road cells, 1 for cells that have a road intersecting
- Homebase-raster: -1 for non-home base cells, household identity number (*p_homebase_id*) for home base cells. Multiple raster files are written if $n_{\mathrm{s,c}} > 1$.
- Ownership-raster: -1 for cells that are not owned, household identity number (*p_owner*) for cells that are owned by a household. Note: inaccessible areas do not show up in this output.
- Land-use-type-raster: 0 for 'others' and road cells (i.e. also home bases), 1, 2, 3, 4, 5 for the different crop types, 1000 for inaccessible areas
- Patch-id-raster: -1 for cells that are not smallholder fields (also inaccessible areas), patch identity number (*p_id*) for smallholder fields
- Matrix-patch-raster: -1 for cells that have a road intersecting and that are not 'others' cells, 'others' patch identity number (*p_matrixcluster_id*) for 'others' cells.

Besides raster output, the write-road-shapefiles procedure creates a polyline-shapefile from the current road network, which is useful if one of the artificial road creation algorithms has been used.

Additionally, EFForTS-LGraf offers an output function that utilizes the 3D renderer of NetLogo, to create a three dimensional illustration of the generated landscape using trees, palms and houses to visualize the different crop types (Fig A1). With little code adjustments, this feature could in general be used to display other crop types as well.

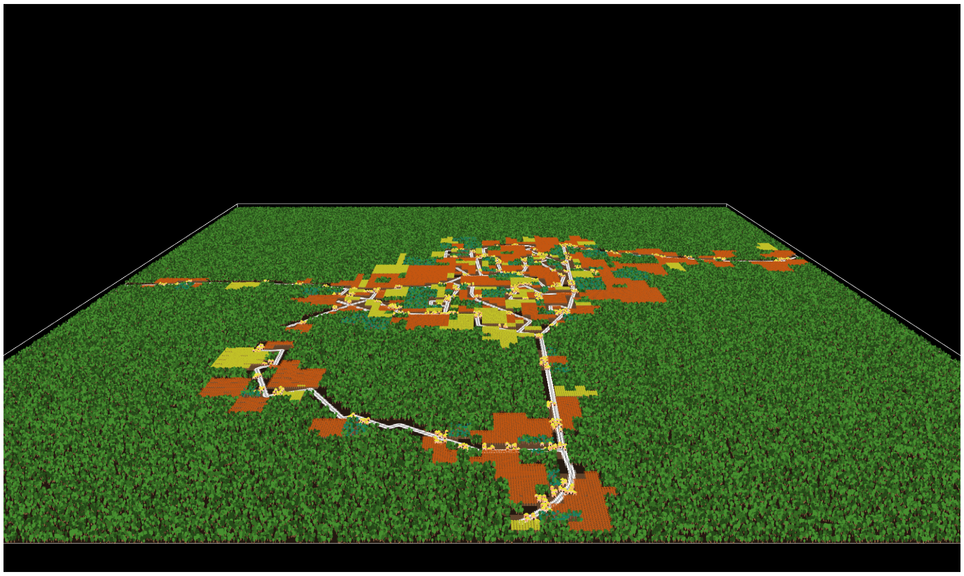

Figure A1: 3d rendered snapshot of EFForTS-LGraf. White lines represent the real road polylines derived from GIS data. ’Others’ cells are illustrated as green shaded tree shapes. Oil palm plantations are illustrated as orange colored palm rows. Rubber plantations are illustrated as yellow colored tree rows. Agroforestry cells are illustrated as mixed rows and trees in blue and green color. Settlements are illustrated by small house symbols.

1.2 Analysis Details

1.2.1 Approach 1: Sobol sensitivity analysis

Table A1: Sobol sensitivity analysis parameter ranges

id	Name on GUI	variation	value
t_s	setup-type	constant	"area"
$n_{s,h}$	number-of-farmers	not in use	-
$n_{s,v}$	number-of-villages	not in use	-
$n_{s,a}$	prop-agricultural-area	uniform	min=0.05, max=0.75
$n_{s,c}$	households-per-cell	uniform (integer)	min=3, max=6
$seed_s$	rnd-seed	set externally	-
rep_s	reproducable?	constant	true
w_s	width	constant	100
h_s	height	constant	100
c_s	cell-length-meter	constant	50
t_r	road-type	constant	"artificial.perlin"
$i_{r,shp}$	road-map-nr	not in use	-
$n_{r,art}$	total-road-length	uniform	min=500, max=1000
$m_{r,art}$	min-dist-roads	constant	5
$p1_{r,perl}$	perlin-octaves	uniform	min=2, max=12
$p2_{r,perl}$	perlin-persistence	uniform	min=0.1, max=0.9
$p3_{r,perl}$	cone-angle	uniform	min=90, max=180
$p4_{r,perl}$	dist-weight	uniform	min=0, max=1
d_v	vlg-size-distribution	constant	"uniform"
μ_v	vlg-size-mean_ha	uniform	min=10, max=20
σ_v	vlg-size-sd_ha	uniform	min=2, max=10
d_v	vlg-min-distance	uniform	min=1, max=10
d_h	hh-area-distribution	constant	"log-normal"
μ_h	hh-area-mean_ha	uniform	min=0.25, max=3
σ_h	hh-area-sd_ha	uniform	min=0.25, max=1
n_i	inaccessible-area-fraction	constant	0
l_i	inaccessible-area-location	not in use	-
d_i	inaccessible-area-distribution	not in use	-
μ_i	inaccessible-area-mean	not in use	-
σ_i	inaccessible-area-sd	not in use	-
t_f	field-type	constant	"percentage"
d_f	field-size-distribution	constant	"normal"
μ_f	field_size_mean_ha	not in use	derived as percentage from hh-area-mean_ha

σ_f	field_size_sd_ha	uniform	min=0.25, max=2
p_f	field-size-percentage	uniform	min=0.1, max=0.9
s_f	field-shape-factor	uniform	min=0.7, max=2
t_{strat}	strategies-type	constant	"id"
$s1_{strat}$	s1.homebase	not in use	derived from field-strategies-id
$s2_{strat}$	s2.fields	not in use	derived from field-strategies-id
$s3_{strat}$	s3.nearby	not in use	derived from field-strategies-id
$s4_{strat}$	s4.avoid	not in use	derived from field-strategies-id
n_{strat}	change-strategy	uniform	min=5, max=20
i_{strat}	field-strategies-id	uniform (integer)	min=1, max=9
t_l	land-use-assignment	constant	"landscape-level-fraction"
id_l	LUT-l-name (l=1,2,3,4,5)	constant	l=1: "fields"; l>1 not in use
fr_l	LUT-l-fraction (l=1,2,3,4,5)	constant	l=1: 1; l>1 not in use
$fr_{l,spec}$	LUT-l-specialize (l=1,2,3,4,5)	not in use	-
f_l	LUT-fill-up	constant	"LUT-1-fraction"

We created a parameter input matrix in order to calculate Sobol Sensitivity Indices for model parameters on different outputs (here: landscape metrics). The parameter matrix was created by variation of parameters in defined ranges (Table A1). The parameter matrix was created with 500 samples and 10 bootstrapping replicates which results in 9500 simulations. For calculating sensitivity indices we used the optimized Sobol estimator proposed by Jansen [4–7].

1.2.2 Approach 2: Validation

To study the range of landscape characteristics that EFForTS-LGraf can create, we performed a second validation approach in addition to the genetic algorithm validation approach (see Approach 2, main text). From the reclassified land-cover map, that was used for the genetic algorithm validation approach, we sampled 100 randomly placed landscapes, 100×100 cells in size (landscapes may overlap), and calculated the five landscape metrics for each of these sampled landscapes and each land-cover type (fields and other). We compared the landscape characteristics (landscape metrics)

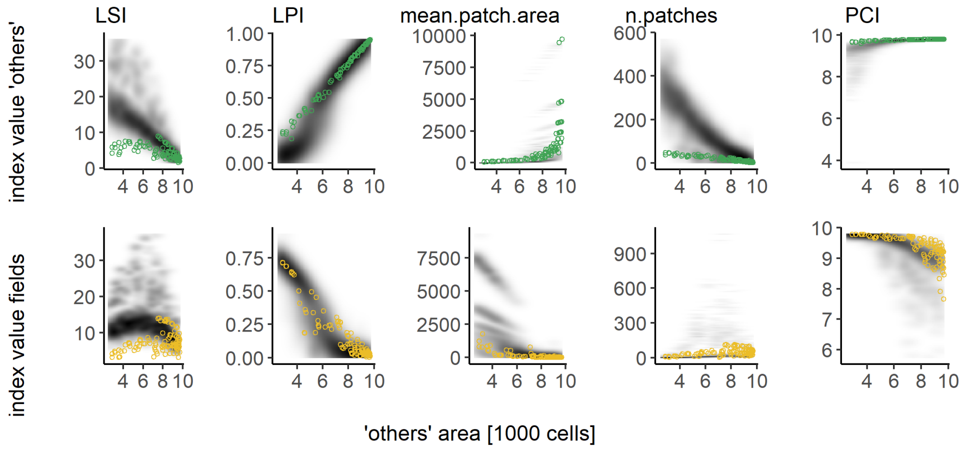

Figure A2: Points indicate distributions of landscape metrics from 100 landscape samples (100 × 100 *cells*) from the re-classified satellite image of the Harapan region. Background shading indicates distributions of landscape metrics from 9500 generated landscapes of the same size and resolution (Sobol sensitivity analysis, approach 1). The darker the shading, the more often a landscape with the corresponding index value has been generated. Index distributions of 'others' cells and patches are presented in the upper row (green points, 'others'), whereas index distributions of field cells and patches are presented in the bottom row (yellow points, fields).

of these landscapes to the 9500 artificially generated landscapes from the Sobol sensitivity analysis (approach 1). To allow comparability, all agricultural crop types within the generated landscapes were aggregated to one class (*fields*) and the remaining area was classified as class 'others'.

Large heterogeneity was found in landscape metrics from 100 randomly placed sample landscapes (100 $\times$ 100 cells) within the classified land-cover map (point distributions in Fig A2). Landscape metrics were calculated as a function of 'others' cells area, because most indices are very sensitive to class proportions. The landscape shape index (LSI) quantifies the spatial aggregation of patches for each land-cover type. Compared to our generated landscapes, the sampled landscapes covered a relatively small range of aggregation levels (Fig A2). For landscapes with small 'others' area, our generated landscapes had higher LSI than the sampled landscapes. The largest patch index was almost linearly correlated with the 'others' area (negative for agricultural patches, positive for 'others' patches). With increasing 'others' area, the mean patch area was exponentially increasing for 'others' patches in both, the generated and the sampled landscapes. For fields, the mean patch area was almost constant at a very low level. Compared to our generated landscapes, the number of 'others' patches was relatively low, especially for landscapes with small 'others' area. The patch cohesion index (PCI) quantifies the shape complexity and perimeter density of patches and showed a decreasing trend for agricultural patches with increasing 'others' area. For 'others' patches, the patch cohesion index was almost constant across the 100 sampled landscapes.

Comparing the 9500 landscapes that were generated within the Sobol sensitivity analysis approach (approach 1) with the 100 realistic landscapes, many characteristics found in the sampled landscapes (point distributions in Fig A2) were covered by our generated landscapes (background shading in Fig A2). The generated landscapes covered a wide range of landscape shape index (LSI) values. However, generated landscapes with small 'others' area showed systematically higher LSI values. The largest patch index (LPI) distribution was matched very accurately and all sampled landscapes lay within the boundaries of the 9500 generated landscapes. EFForTS-LGraf was also able to reproduce a wide range of mean patch area although the observed heterogeneity in the sampled landscapes was significantly lower. The variety of the total number of patches was reproduced for field patches, but did not fit well for 'others' patches, especially when 'others' area was small. For the patch cohesion index all sampled landscapes lay within the boundaries of our generated landscapes.

In order to visualize the similarity between sampled and generated land-

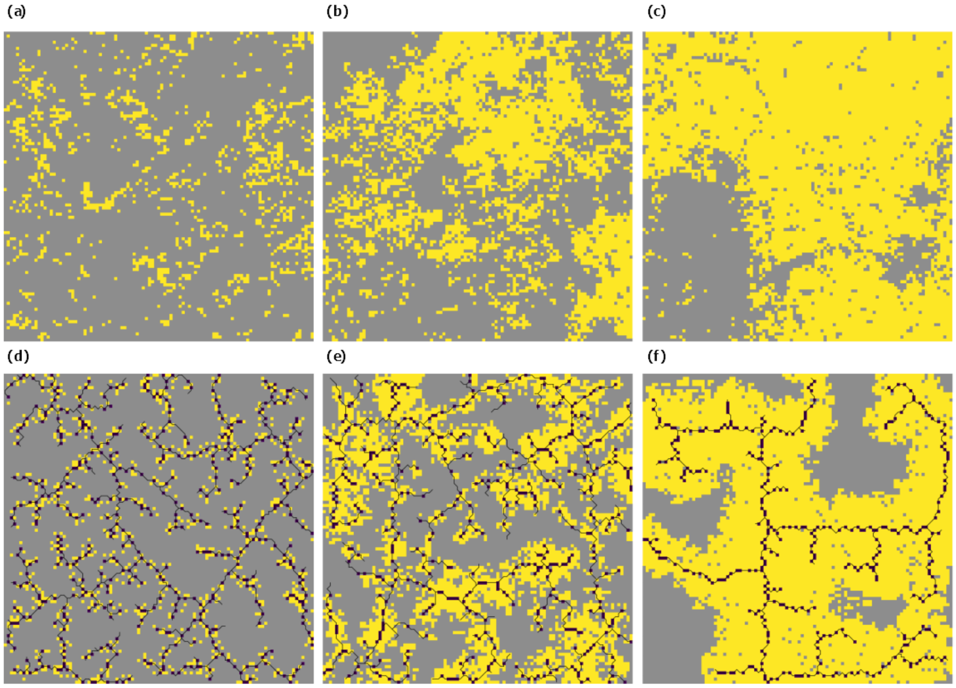

Figure A3: Three of the 100 sampled landscapes from the re-classified satellite image (a, b, c) and three generated EFForTS-LGraf landscapes from the Sobol sensitivity analysis (d, e, f). Grey cells indicate 'others' cells and yellow cells indicate fields. For the EFForTS-LGraf landscapes, black cells indicate household home bases and lines indicate artificially generated roads.

cover maps, we selected three different example landscapes with different 'others' cells area from the 100 samples of the Harapan land-cover map and three example landscapes from the 9500 generated artificial landscapes with matching 'others' area (Fig A3). By visual comparison, the resulting landscapes from EFForTS-LGraf showed similar spatial clustering of patches and patch sizes although the landscapes from the Harapan land-cover map contained slightly more small field patches that were slightly more scattered across the 'others' area. This matched the observation from the landscape metrics comparison, where the sampled landscapes from the classified land-use map had a significantly greater number of field patches compared to our generated landscapes (Fig A3, n patches).

1.2.3 Approach 3: Applied case study

Table A2: Applied case study parameter ranges

**id**	**Name on GUI**	**variation**	**value**
t_{s}	setup-type	constant	"area"
$n_{\mathrm{s,h}}$	number-of-farmers	not in use	-
$n_{\mathrm{s,v}}$	number-of-villages	not in use	-
$n_{\mathrm{s,a}}$	prop-agricultural-area	constant	0.5
$n_{\mathrm{s,c}}$	households-per-cell	constant	1
$seed_{\mathrm{s}}$	rnd-seed	set externally	-
rep_{s}	reproducable?	constant	true
w_{s}	width	constant	100
h_{s}	height	constant	100
c_{s}	cell-length-meter	constant	50
t_{r}	road-type	constant	"real.shapefile"
$i_{\mathrm{r,shp}}$	road-map-nr	constant	3
$n_{\mathrm{r,art}}$	total-road-length	not in use	-
$m_{\mathrm{r,art}}$	min-dist-roads	not in use	-
$p1_{\mathrm{r,perl}}$	perlin-octaves	not in use	-
$p2_{\mathrm{r,perl}}$	perlin-persistence	not in use	-
$p3_{\mathrm{r,perl}}$	cone-angle	not in use	-
$p4_{\mathrm{r,perl}}$	dist-weight	not in use	-
d_{v}	vlg-size-distribution	constant	"uniform"
μ_{v}	vlg-size-mean_ha	constant	15
σ_{v}	vlg-size-sd_ha	constant	6
d_{v}	vlg-min-distance	constant	10
d_{h}	hh-area-distribution	constant	"log-normal"
μ_{h}	hh-area-mean_ha	uniform	min=1, max=3
σ_{h}	hh-area-sd_ha	constant	0.91

n_{i}	inaccessible-area-fraction	constant	0
l_{i}	inaccessible-area-location	not in use	-
d_{i}	inaccessible-area-distribution	not in use	-
μ_{i}	inaccessible-area-mean	not in use	-
σ_{i}	inaccessible-area-sd	not in use	-
t_{f}	field-type	constant	"distribution"
d_{f}	field-size-distribution	constant	"log-normal"
μ_{f}	field_size_mean_ha	constant	0.49
σ_{f}	field_size_sd_ha	constant	0.77
p_{f}	field-size-percentage	not in use	-
s_{f}	field-shape-factor	constant	1
t_{strat}	strategies-type	constant	"id"
$s1_{\mathrm{strat}}$	s1.homebase	not in use	derived from field-strategies-id
$s2_{\mathrm{strat}}$	s2.fields	not in use	derived from field-strategies-id
$s3_{\mathrm{strat}}$	s3.nearby	not in use	derived from field-strategies-id
$s4_{\mathrm{strat}}$	s4.avoid	not in use	derived from field-strategies-id
n_{strat}	change-strategy	constant	10
i_{strat}	field-strategies-id	constant	1
t_{l}	land-use-assignment	constant	"household-level-specialization"
id_{l}	LUT-l-name (l=1,2,3,4,5)	constant	l=1: "oilpalm"; l=2: "rubber"; l>2 not in use
fr_{l}	LUT-l-fraction (l=1,2,3,4,5)	constant	l=1: 0.5; l=2: 0.5; l>2 not in use
$fr_{\mathrm{l,spec}}$	LUT-l-specialize (l=1,2,3,4,5)	uniform/constant	l=1: min=0, max=1; l=2: 0, l>2 not in use
f_{l}	LUT-fill-up	constant	"LUT-2-fraction"

We created a parameter input matrix in order to calculate effects of household consolidation and household level specialization on different outputs (here: landscape metrics). The parameter matrix was created by variation of the two parameters *household-area-mean_ha* and *LUT-1-specialize* (Table A2). We generated the parameter matrix by using a Latin Hypercube sampling design with 500 samples [8]. We simulated three repetitions of the parameter matrix to account for stochastic effects. We analyzed the resulting landscapes via the five selected landscape metrics from previous

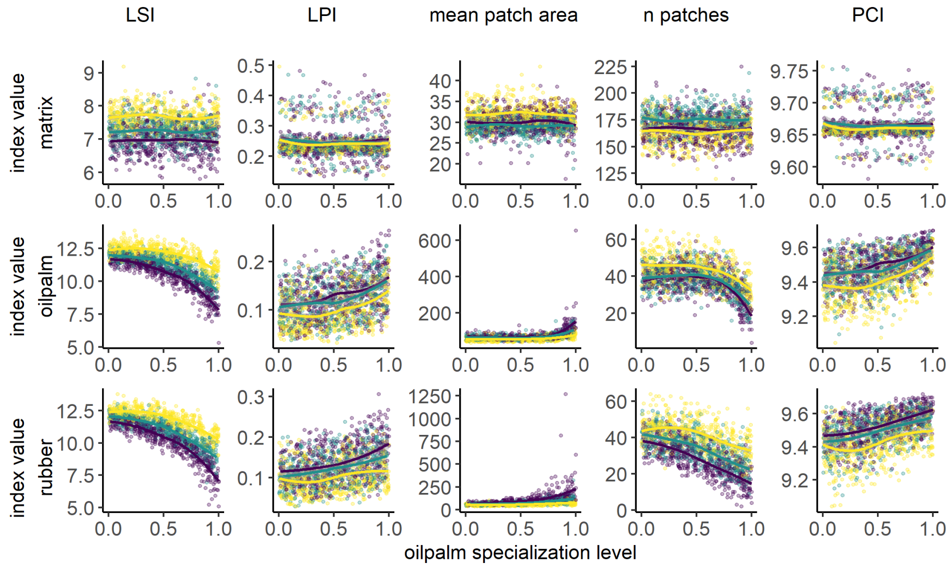

Figure A4: Five selected landscape metrics, for two crop types (oil palm and rubber) and 'others' patches (matrix) calculated for land-cover maps generated by the model using a latin hypercube sampling approach. Plots show the value of the landscape metrics versus household specialization level on oil palm. Differences in household size distributions are shown by colours with darker colors indicating larger household size. These are grouped into small (1 - 1.65 ha, yellow), medium (1.66 - 2.32 ha, green) and large (2.32 - 3 ha, purple). Lines show smoothed trends and standard error using the locally weighted scatterplot smoothing (LOESS) method.

approaches. We calculated linear regression models for each landscape metric and crop type combination and calculated standardized regression coefficients to estimate parameter and interaction effects on landscape metrics of the generated landscapes (coefficient results, see main text). Additionally, we investigated the raw data by plotting landscape metrics as a function of specialization level, grouped by household size (see Fig A4).

References

1. Perlin K, Ken, Perlin, Ken. An image synthesizer. In: Proceedings of the 12th annual conference on Computer graphics and interactive techniques - SIGGRAPH '85. vol. 19. New York, New York, USA: ACM Press; 1985. p. 287–296. Available from: http://portal.acm.org/citation.cfm?doid=325334.325247.

2. Ebert DS, Musgrave FK, Peachey D, Perlin K, Worley S, Mark WR, et al. Texturing & modeling : a procedural approach. Morgan Kaufmann; 2003. Available from: https://nyuscholars.nyu.edu/en/publications/texturing-and-modeling-a-procedural-approach-third-edition.

3. Pe'er G, Zurita GA, Schober L, Bellocq MI, Strer M, Müller M, et al. Simple Process-Based Simulators for Generating Spatial Patterns of Habitat Loss and Fragmentation: A Review and Introduction to the G-RaFFe Model. PLoS ONE. 2013;8(5):e64968. doi:10.1371/journal.pone.0064968.

4. Sobol IM. On sensitivity estimation for nonlinear mathematical models. Matem Mod. 1990;2(1):112–118. doi:10.1029/94JC00847.

5. Saltelli A, Annoni P, Azzini I, Campolongo F, Ratto M, Tarantola S. Variance based sensitivity analysis of model output. Design and estimator for the total sensitivity index. Computer Physics Communications. 2010;181(2):259–270. doi:10.1016/J.CPC.2009.09.018.

6. Jansen MJW. Analysis of variance designs for model output. Computer Physics Communications. 1999;117(1-2):35–43. doi:10.1016/S0010-4655(98)00154-4.

7. Chan K, Tarantola S, Saltelli A, Sobol IM. Variance-Based Methods. In: Saltelli A, Chan K, Scott E, editors. Sensitivity Analysis. Chichester: Wiley; 2000. p. 167–197.

8. McKay MD, Beckman RJ, Conover WJ. A Comparison of Three Methods for Selecting Values of Input Variables in the Analysis of Output from a Computer Code. Technometrics. 1979;21(2):239. doi:10.2307/1268522.
